# A Rapid, Mobile Neurocognitive Screening Test to Aid in Identifying Cognitive Impairment and Dementia (BrainCheck): Cohort Study

**DOI:** 10.2196/12615

**Published:** 2019-03-21

**Authors:** Samantha Groppell, Karina M Soto-Ruiz, Benjamin Flores, William Dawkins, Isabella Smith, David M Eagleman, Yael Katz

**Affiliations:** 1 School of Public Health The University of Texas Health Science Center at Houston Houston, TX United States; 2 BrainCheck Inc Houston, TX United States; 3 Department of Psychiatry and Behavioral Sciences Stanford University School of Medicine Stanford, CA United States

**Keywords:** dementia, neurocognitive tests, neurocognitive computerized assessment tools (NCAT), mild cognitive impairment (MCI), BrainCheck, digital testing, Alzheimer’s disease, electronic neurocognitive tools, computerized cognitive assessment, digital cognitive assessment

## Abstract

**Background:**

The US population over the age of 65 is expected to double by the year 2050. Concordantly, the incidence of dementia is projected to increase. The subclinical stage of dementia begins years before signs and symptoms appear. Early detection of cognitive impairment and/or cognitive decline may allow for interventions to slow its progression. Furthermore, early detection may allow for implementation of care plans that may affect the quality of life of those affected and their caregivers.

**Objective:**

We sought to determine the accuracy and validity of BrainCheck Memory as a diagnostic aid for age-related cognitive impairment, as compared against physician diagnosis and other commonly used neurocognitive screening tests, including the Saint Louis University Mental Status (SLUMS) exam, the Mini-Mental State Examination (MMSE), and the Montreal Cognitive Assessment (MoCA).

**Methods:**

We tested 583 volunteers over the age of 49 from various community centers and living facilities in Houston, Texas. The volunteers were divided into five cohorts: a normative population and four comparison groups for the SLUMS exam, the MMSE, the MoCA, and physician diagnosis. Each comparison group completed their respective assessment and BrainCheck Memory.

**Results:**

A total of 398 subjects were included in the normative population. A total of 84 participants were in the SLUMS exam cohort, 51 in the MMSE cohort, 35 in the MoCA cohort, and 18 in the physician cohort. BrainCheck Memory assessments were significantly correlated to the SLUMS exam, with coefficients ranging from .5 to .7. Correlation coefficients for the MMSE and BrainCheck and the MoCA and BrainCheck were also significant. Of the 18 subjects evaluated by a physician, 9 (50%) were healthy, 6 (33%) were moderately impaired, and 3 (17%) were severely impaired. A significant difference was found between the severely and moderately impaired subjects and the healthy subjects (*P*=.02). We derived a BrainCheck Memory composite score that showed stronger correlations with the standard assessments as compared to the individual BrainCheck assessments. Receiver operating characteristic (ROC) curve analysis of this composite score found a sensitivity of 81% and a specificity of 94%.

**Conclusions:**

BrainCheck Memory provides a sensitive and specific metric for age-related cognitive impairment in older adults, with the advantages of a mobile, digital, and easy-to-use test.

**Trial Registration:**

ClinicalTrials.gov NCT03608722; https://clinicaltrials.gov/ct2/show/NCT03608722 (Archived by WebCite at http://www.webcitation.org/76JLoYUGf)

## Introduction

As the baby boom generation grows older, the percentage of the US population over the age of 65 is expected to double by the year 2050 [1]. Concordantly, by 2030 the incidence of dementia is projected to increase from 35 million to 70 million [2]. Mild cognitive impairment (MCI) is considered an intermediate state between normal age-related decline and dementia. Data from the Mayo Clinic Study of Aging estimate the development of MCI in up to 29% of older individuals during the span of the 5-year longitudinal study [3]. MCI may progress to dementia or represent a potentially reversible condition related to a variety of conditions, including polypharmacy, depression, and sleep apnea [4].

The subclinical stage of dementia begins years before signs and symptoms appear [5]. Once clinically manifested, treatment for dementia is either palliative in nature or aimed at slowing progression, as no curative therapy currently exists [6]. Early detection of cognitive impairment, on the other hand, may identify treatable and reversible conditions. Although reversing disease expression of neurodegenerative conditions such as Alzheimer’s disease is not possible at this time, early detection of cognitive decline may allow for interventions to slow its progression or for implementation of care plans that may impact the quality of life of affected individuals and their caregivers [7].

The most commonly used neurocognitive screening tests include the Saint Louis University Mental Status (SLUMS) exam [8], the Mini-Mental State Examination (MMSE) [9], and the Montreal Cognitive Assessment (MoCA) [10]. These tools are able to distinguish impaired individuals from their healthy counterparts. Recent studies have reported the diagnostic sensitivity and specificity of the MMSE to be 81% and 89%, respectively [11], with similar performance for the SLUMS exam (82% and 86%, respectively), and the MoCA (91% and 81%, respectively) [11,12].

Although commonly used in clinical practice, none of the methods noted above are considered the “gold standard” for cognitive screening [13]. While the MMSE, SLUMS exam, and MoCA have relatively high sensitivities and specificities, each screener contains shortcomings. The MMSE relies heavily on memory and language, with little emphasis on other cognitive domains, such as executive function and visuospatial attention [14]. The SLUMS exam includes tests of executive function but is inferior to the MMSE when assessing activities of daily living and functionality [15]. The MoCA appears to be the most robust screener, however, it requires more research to establish its validity [16].

Furthermore, these screening tools are verbally administered by a physician or test administrator, with responses and scores recorded with pen and paper. When integrated into a physician assessment, the tools may be time-consuming, and the need for a test administrator may increase expenses but adds no additional physician reimbursement [17]. While the screening instruments are relatively simple to administer, it is uncertain whether the instruments are commonly administered and scored as intended in routine clinical practice. For example, a European study reported significant score discrepancies between MMSEs performed by general practitioners and neuropsychologists [18]. Digital neurocognitive testing has several advantages that include the following: (1) elimination of potential practice effects [19] and floor or ceiling effects [20] typically seen in pen-and-paper versions, (2) automated administration and scoring of the test items, and (3) automatic integration with electronic medical records [21]. In addition, digital testing can be readily delegated to a technician, thus focusing the clinician’s time on interpretation and decision making rather than test administration and scoring.

BrainCheck Sport is a computerized neurocognitive test available on iPad, iPhone, or a desktop browser and was previously validated for its diagnostic accuracy for the detection of concussion [22]. BrainCheck Memory is a modified version of this program that targets dementia-related cognitive decline. BrainCheck Memory functions as an app that can be downloaded from the Apple Store and accessed via password-protected log-in. The primary aim of this study was to assess the utility and accuracy of BrainCheck Memory—herein referred to as BrainCheck or BrainCheck Memory—as a computerized diagnostic tool for cognitive impairment among older adults.

## Methods

This study of 583 subjects was subdivided into five cohorts for analyses: a normative population; SLUMS exam, MMSE, and MoCA comparison groups; and a physician-diagnosis comparison group. Additionally, a composite score was calculated to provide a sensitive metric for cognitive impairment.

### Normative Population

Participants were volunteers from community centers, assisted living facilities, and a church in Houston, Texas. Inclusion criteria were as follows: age greater than or equal to 50 years, function in at least one hand, and normal or corrected vision. Exclusion criteria included a history of stroke or other neurological disability (eg, attention deficit hyperactivity disorder [ADHD] or epilepsy), inability to speak English or Spanish, and illiteracy, defined for study purposes as unable to read the written informed consent. All participants signed informed consent forms prior to participation in the study, as approved by the Solutions Institutional Review Board. No compensation was provided for study participation.

All testing was completed on iPads or iPhones. Tests were administered by trained, bilingual research staff and performed one-on-one in a quiet, well-lit space. Participants were provided with brief instructions prior to taking the battery of assessments, and clarification was provided during testing if needed. Additional instructions were not provided once testing began.

### Comparison to Reference Screening Methods

Volunteers for the SLUMS exam and MMSE comparison groups were recruited via convenience sampling from community centers; volunteers for the MoCA and physician groups were recruited from two assisted-living facilities.

Diagnostic performance of BrainCheck was compared to that of an electronic version of the SLUMS exam created for this research. Prior to conducting BrainCheck’s assessments, research staff administered the SLUMS exam via a Wi-Fi-connected iPad or iPhone. After completing the SLUMS exam, participants completed the BrainCheck assessment on the same device used during the SLUMS exam administration. Subjects with scores of 20 or lower on the SLUMS exam were included in the dementia group and those with scores of 21 or higher in the control group [8].

Screening performance of BrainCheck was also compared to both pen-and-paper versions of the MMSE and the MoCA. Pen-and-paper testing was performed before BrainCheck, which was administered on either an iPad or iPhone.  

Finally, BrainCheck’s effectiveness as a screening tool was compared to physician diagnosis. A licensed psychiatrist and medical adjudicator evaluated a sample of residents from two separate assisted-living facilities. Evaluations were performed one-on-one in a private space after the participant completed BrainCheck. While the psychiatrist and medical adjudicator provided evaluations following BrainCheck administration, BrainCheck results were not accessible to the practitioners during the course of the evaluation. Physician diagnosis was based on a personal and medical history followed by administration of the MoCA test. Volunteers were diagnosed as healthy, moderately impaired, or severely impaired.

### Description of BrainCheck Battery

Identification of dementia requires impairment of at least two of the following domains: memory, language, praxis, gnosis, or executive functioning [23]. As such, BrainCheck Memory is a compilation of seven neurocognitive tests based on commonly included instruments in neuropsychological test batteries for detection of cognitive impairment. Six of BrainCheck Sport’s assessments—Immediate and Delayed Recall, the Trail Making Test (TMT) A, the Trail Making Test B, the Stroop Test, and the Digit Symbol Substitution Task [22]—are included in BrainCheck Memory. Additionally, the Matrix Problems Task, adapted from the Raven Standard Matrices Test, was added to the battery of assessments to measure fluid intelligence (ie, the ability to reason and problem solve), a skill that commonly declines with age [24]. Participants were shown a pattern of three shapes and asked to select the next shape in the pattern series by choosing from six possibilities. Previous studies showed that dementia patients correctly identify a lesser proportion of matrices compared to elderly controls [25].  

## Results

### Normative Data

**Figure 1:**
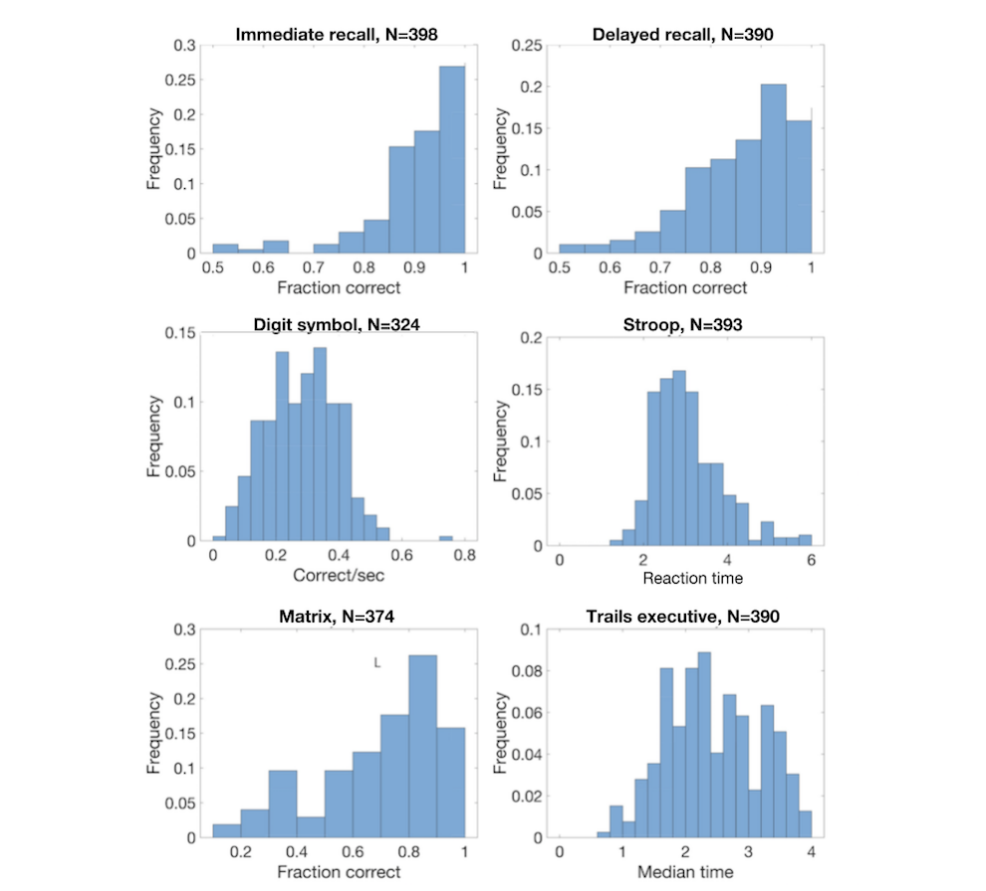
Normative distribution. Distributions of scores for individuals in the normative population are shown for each assessment. The number of normative data points in each distribution is indicated above each panel.

We obtained normative data for 398 participants aged 50-91 years. Data were collected between November 19, 2015, and August 16, 2017. This population consisted of 318 (79.9%) female and 80 (20.1%) male participants. Gender distribution of subjects, while skewed compared to the general population, was determined by voluntary enrollment patterns in the study settings. The mean age was 70.2 years (SD 9.0). Distributions of scores for each assessment are shown in Figure 1, and basic statistics are shown in Table 1. All distributions were unimodal.

### Comparison With the Saint Louis University Mental Status Exam

**Figure 2:**
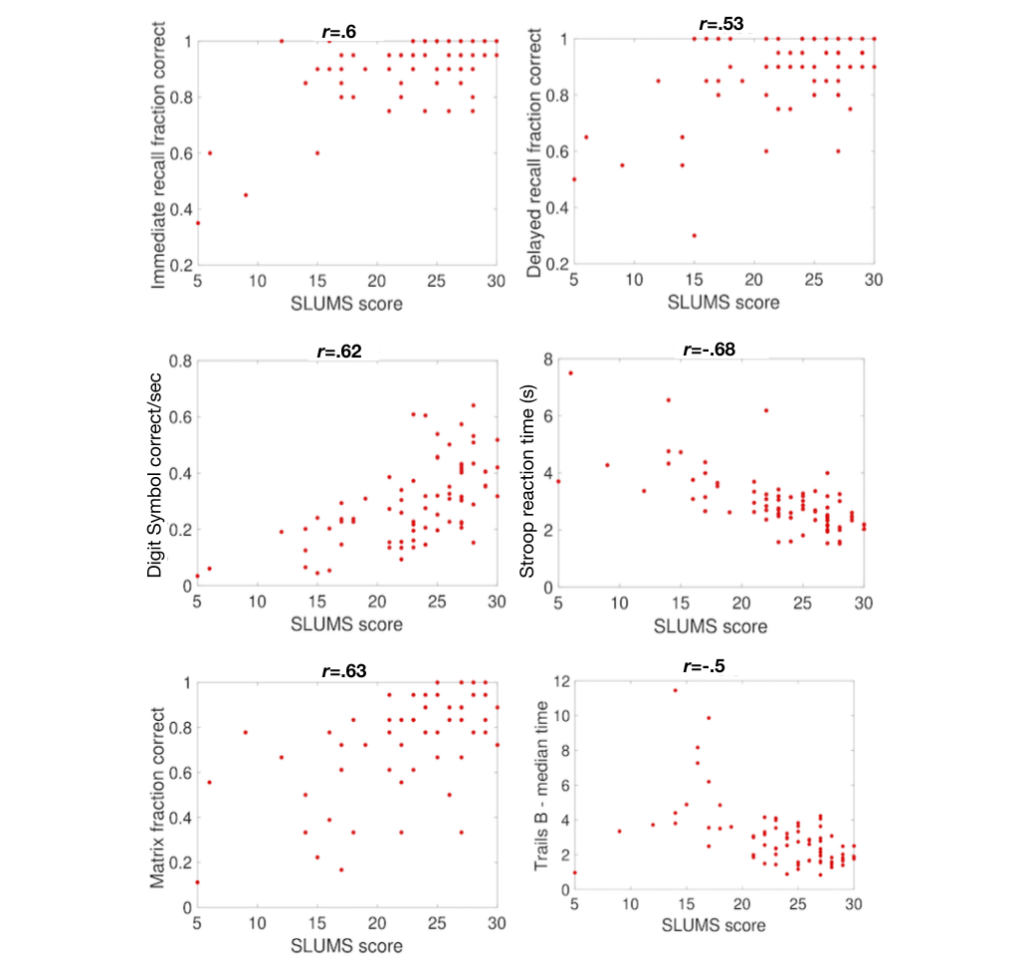
Comparison of BrainCheck assessments with the Saint Louis University Mental Status (SLUMS) exam. Shown are comparisons between SLUMS scores and the scores for each assessment. Each data point represents one participant who took both assessments. Pearson correlation coefficients are indicated above each panel.

A total of 84 subjects were enrolled between November 22, 2016, and August 16, 2017. Of these, 19 (23%) were classified as demented—17 (89%) female; mean age 75 years (SD 9.5). These subjects were compared to 65 controls—55 (85%) female; mean age 62.9 years (SD 16.5). BrainCheck assessments correlated to SLUMS exam scores are shown in Figure 2. Analysis also revealed that BrainCheck batteries span a range of difficulties and domains that influence their correlation with the SLUMS test. For example, while most participants with a SLUMS exam score above 20 were able to perform equally well on the TMTs, the Digit Symbol Substitution Task effectively distinguished between participants in this range. Thus, the TMTs are easier than the Digit Symbol Substitution Task and may be better at detecting dementia while the Digit Symbol Substitution Task may be better at detecting milder cognitive impairments.

**Table 1:** Basic statistics of assessments used in the BrainCheck Memory battery.

Metric	Mean (SD)
Immediate recall fraction (%) correct	94 (7)
Delayed recall fraction (%) correct	91 (9)
Stroop mean reaction time in seconds	2.28 (0.74)
Trails A median reaction time in seconds	1.05 (0.44)
Trails B median reaction time in seconds	1.96 (0.98)
Matrix fraction (%) correct	83 (0.18)
Digit Symbol mean number correct per second	0.44 (0.14)

### Comparison With the Mini-Mental State Examination

**Figure 3:**
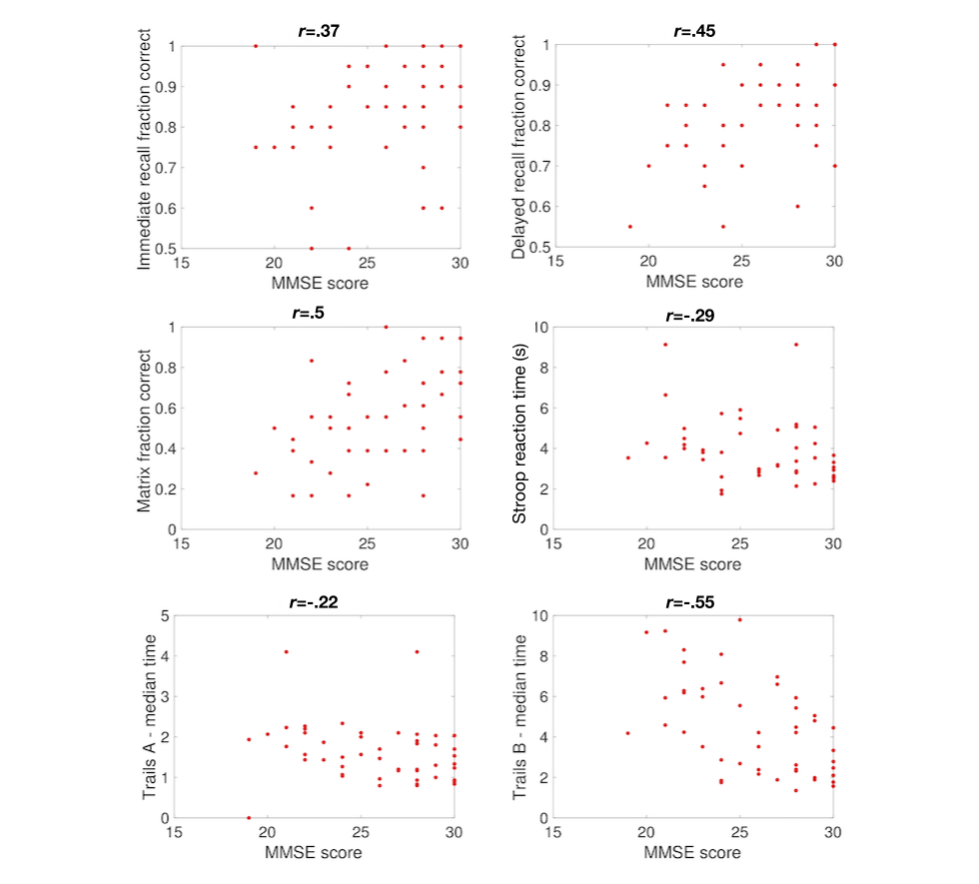
Comparison of BrainCheck assessments with the Mini-Mental State Examination (MMSE). Shown are comparisons between MMSE scores and the scores for each assessment. Each data point represents one participant who took both assessments. Pearson correlation coefficients are indicated above each panel.

Subjects who took the MMSE and BrainCheck (n=51) had a mean age of 73 years (SD 8.3), and 44 (86%) were female. Correlation coefficients between individual BrainCheck assessments and the MMSE were typically lower than with the SLUMS exam, but all were statistically significant and ranged in magnitude from .2 to .55 (see Figure 3).

### Comparison With the Montreal Cognitive Assessment

**Figure 4:**
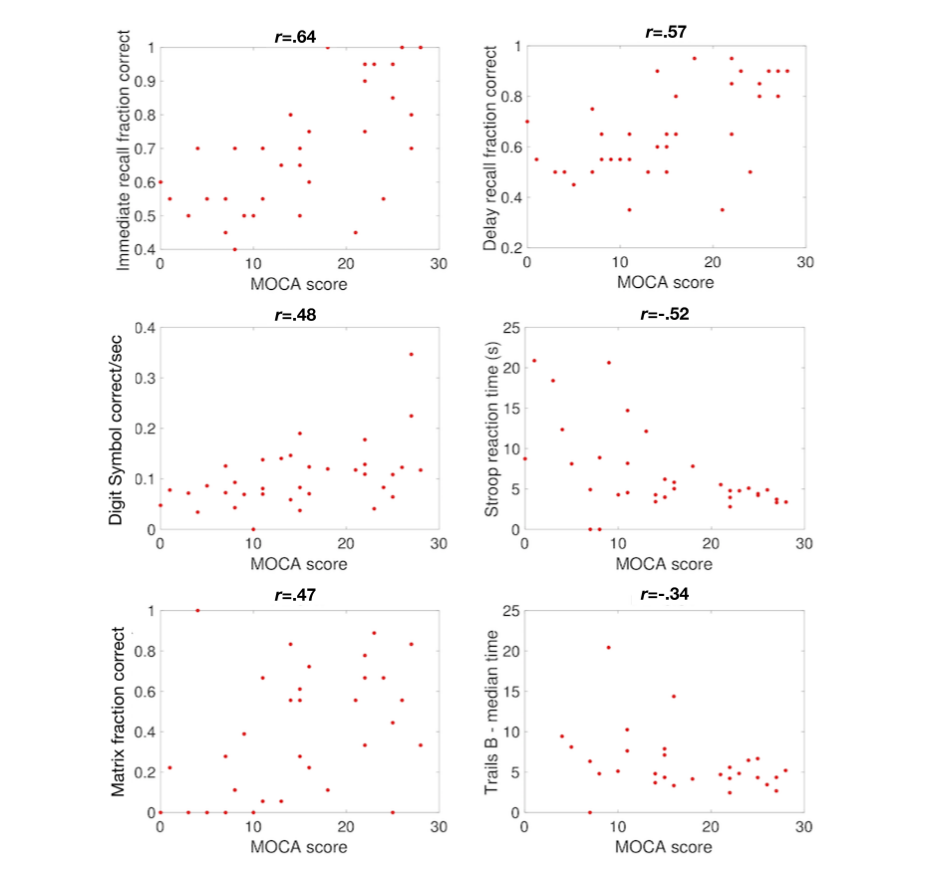
Comparison of BrainCheck assessments with the Montreal Cognitive Assessment (MoCA). Shown are comparisons between MoCA scores and the scores for each assessment. Each data point represents one participant who took both assessments. Pearson correlation coefficients are indicated above each panel.

Of subjects taking the MoCA and BrainCheck (n=35), the mean age was 85.2 (SD 6.3) and 30 (86%) were female. All BrainCheck assessments had correlation coefficients from .3 to .64 (see Figure 4).

### Comparison With Physician Evaluation

**Figure 5:**
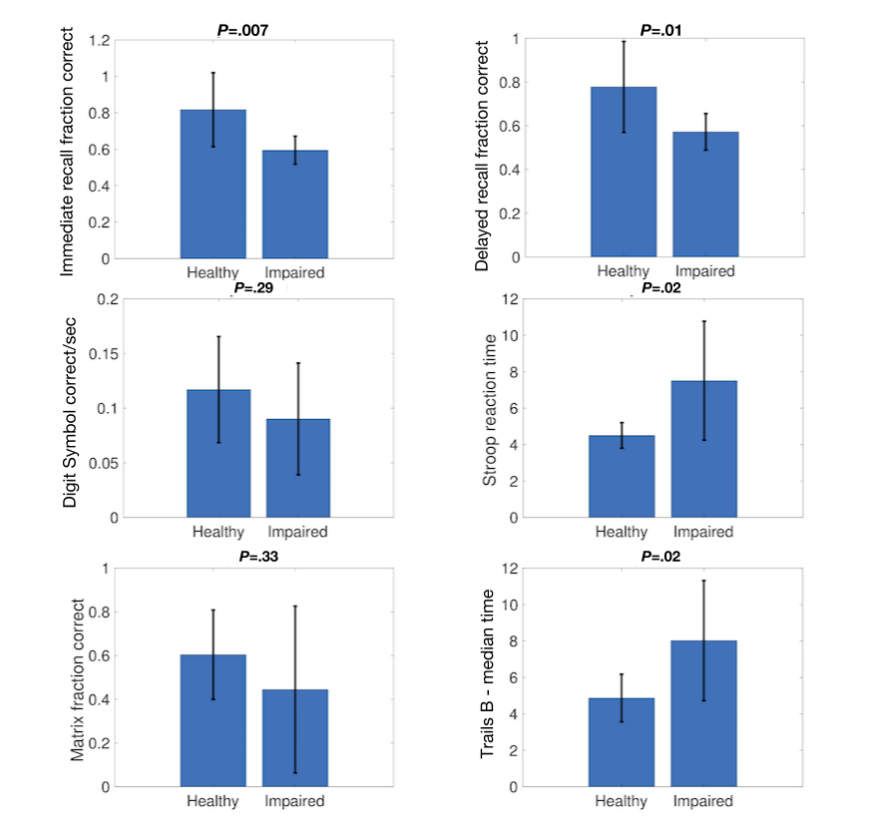
Comparison of BrainCheck assessments with physician diagnosis. Shown are mean scores on each assessment for patients classified as healthy or impaired by a physician. *P* values determined by a two-sided *t* test are given above each panel.

A total of 18 subjects underwent physician evaluation: the mean age was 85.9 years (SD 7.3), 9 (50%) were healthy, 6 (33%) were judged to be moderately impaired, and 3 (17%) were judged to be severely impaired. Comparing the 9 moderately or severely impaired subjects to the controls, we found that 4 out of 6 (67%) BrainCheck assessments identified significant differences (*P*=.02) between the populations (see Figure 5), while the other two showed nonsignificant differences, possibly due to the small sample size.

### Defining a Composite Score for the BrainCheck Battery

**Figure 6:**
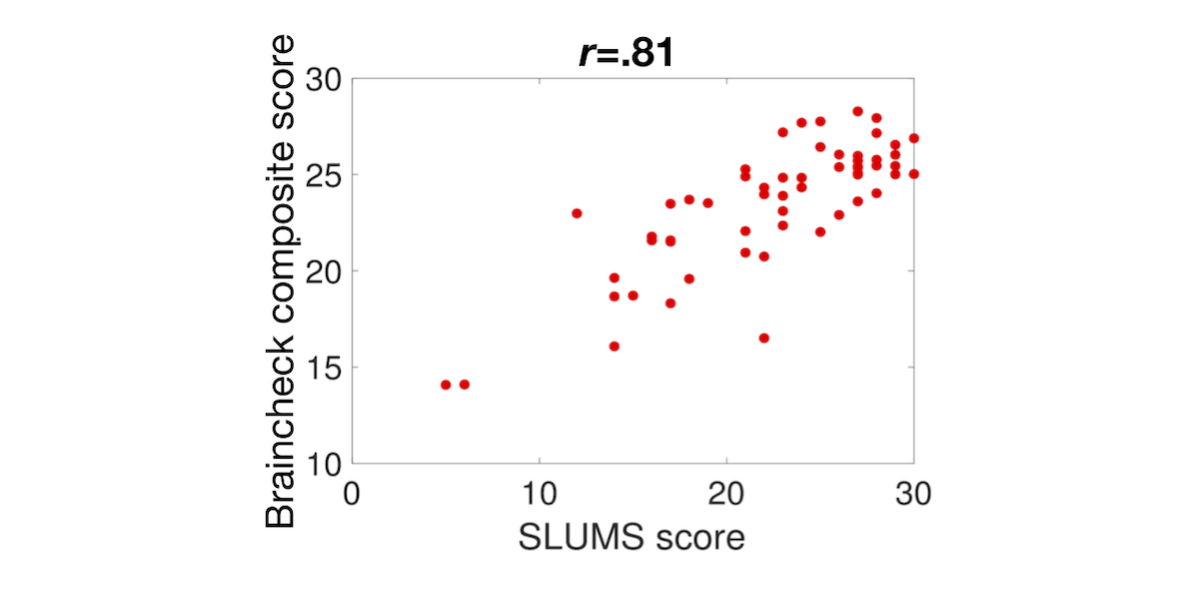
Comparison between BrainCheck composite score and the Saint Louis University Mental Status (SLUMS) exam.

We defined a scaled score for each assessment (*s*_*a*_), such that it fell between 0 and 1. We then defined each assessment’s contribution to the composite score (*c*_*a*_) as *c*_*a*_= *w*_*a*_*s*_*a*_ in assessments with metrics where higher scores indicated higher performance, such as the fraction of correct answers, and *c*_*a*_= *w*_*a*_(1- *s*_*a*_) in cases where higher scores indicated worse performance, such as in tests that measure a reaction time. The weights (*w*_*a*_) were scaled such that their sum was 30, which ensures all composite scores fall between 0 and 30 per other established metrics, such as the SLUMS exam and MMSE. We then used an optimization algorithm to optimize the weights (*w*_*a*_) to maximize the correlation between the composite BrainCheck score and the score on the SLUMS test. Once defined, we applied this optimized metric to our normative population and found a mean of 22.2 with a standard deviation of 2.9. With this optimized metric, we found excellent correlation between the BrainCheck score and the SLUMS exam score—Pearson correlation coefficient, *r*=.81 (see Figure 6).

**Figure 7:**
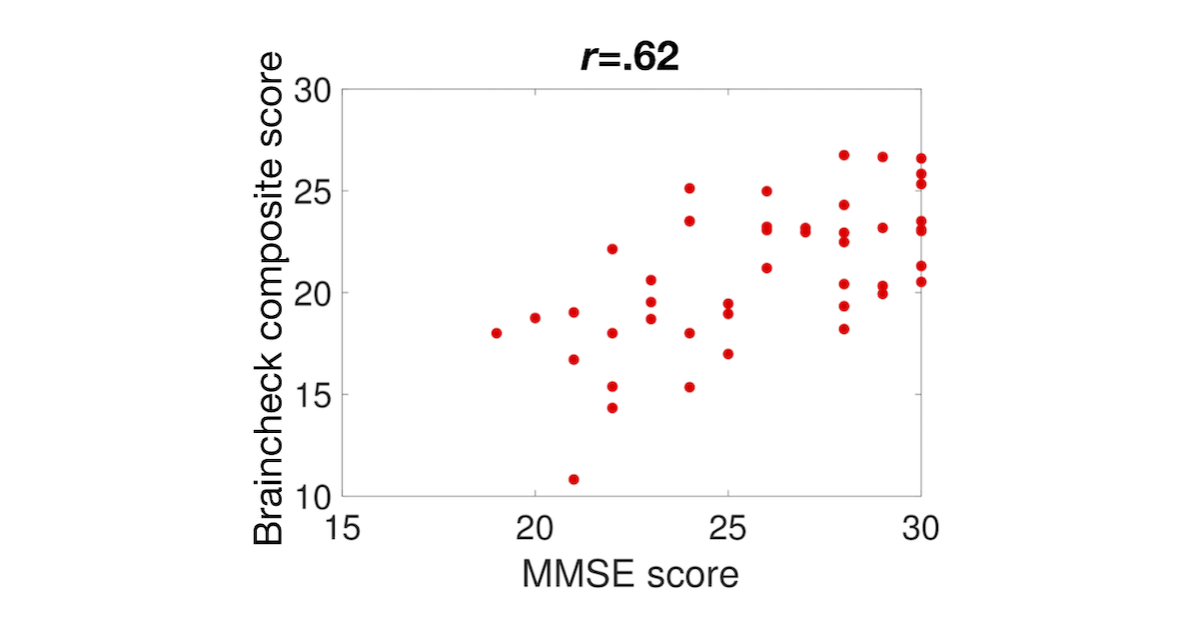
Comparison between BrainCheck composite score and the Mini-Mental State Examination (MMSE).

**Figure 8:**
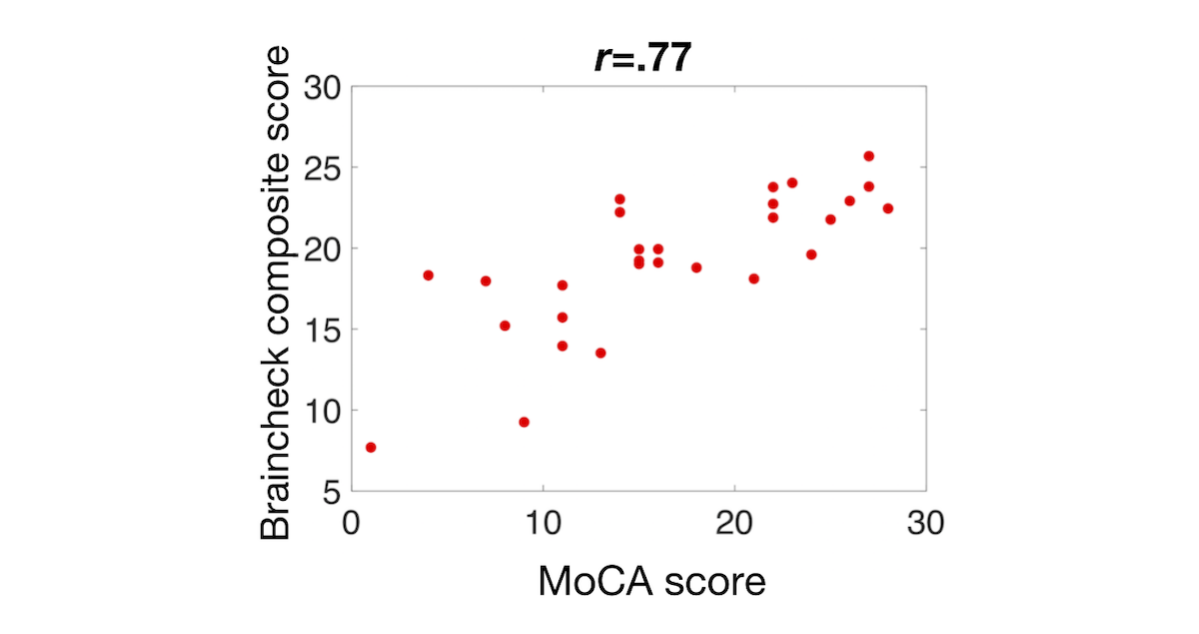
Comparison between BrainCheck composite score and the Montreal Cognitive Assessment (MoCA).

To verify that this composite score performs well against other screening methods that were not used in the optimization, we evaluated the optimized composite score against the MMSE. We again found a strong correlation between the BrainCheck composite score and the MMSE score—Pearson correlation coefficient, *r*=.62 (see Figure 7)—which was stronger than both the correlations of the MMSE with any of the individual assessments and the correlation with the average of the BrainCheck assessments (*r*=.44). We further compared the composite score with the MoCA and found the composite score to outperform each of the individual assessments—Pearson correlation coefficient, *r*=.77 (see Figure 8).

We compared the BrainCheck composite scores in the groups of healthy and impaired individuals as measured by physician diagnosis. We found that impaired individuals had mean BrainCheck composite scores of 14.4 (SD 3.8) as compared to 20.4 (SD 2.2) in the healthy individuals, a highly significant difference (*P*<.001). We noted that the mean score in the group diagnosed as healthy by the physician was still below the mean of our normative population, potentially indicating BrainCheck’s ability to detect subtler cognitive deficits than a binary diagnosis.

**Figure 9:**
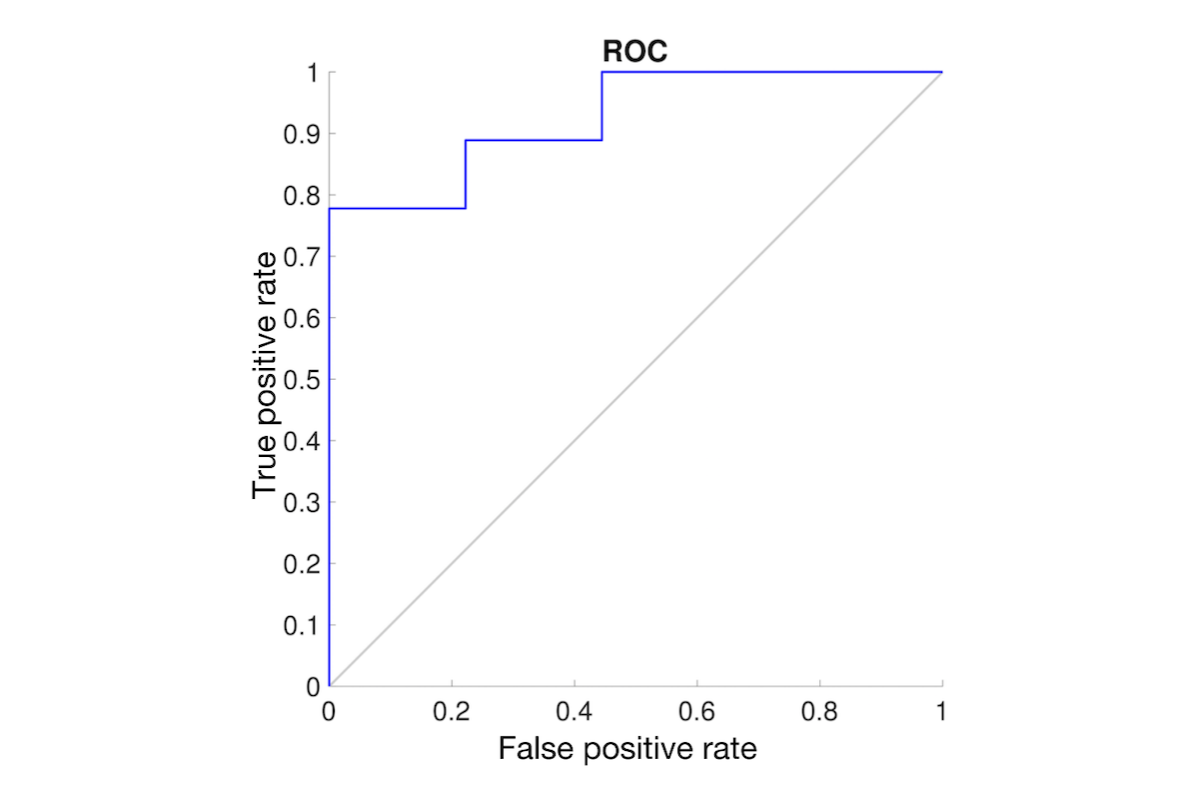
Receiver operating characteristic (ROC) curve for comparison between the physician diagnosis and the BrainCheck composite score.

**Figure 10:**
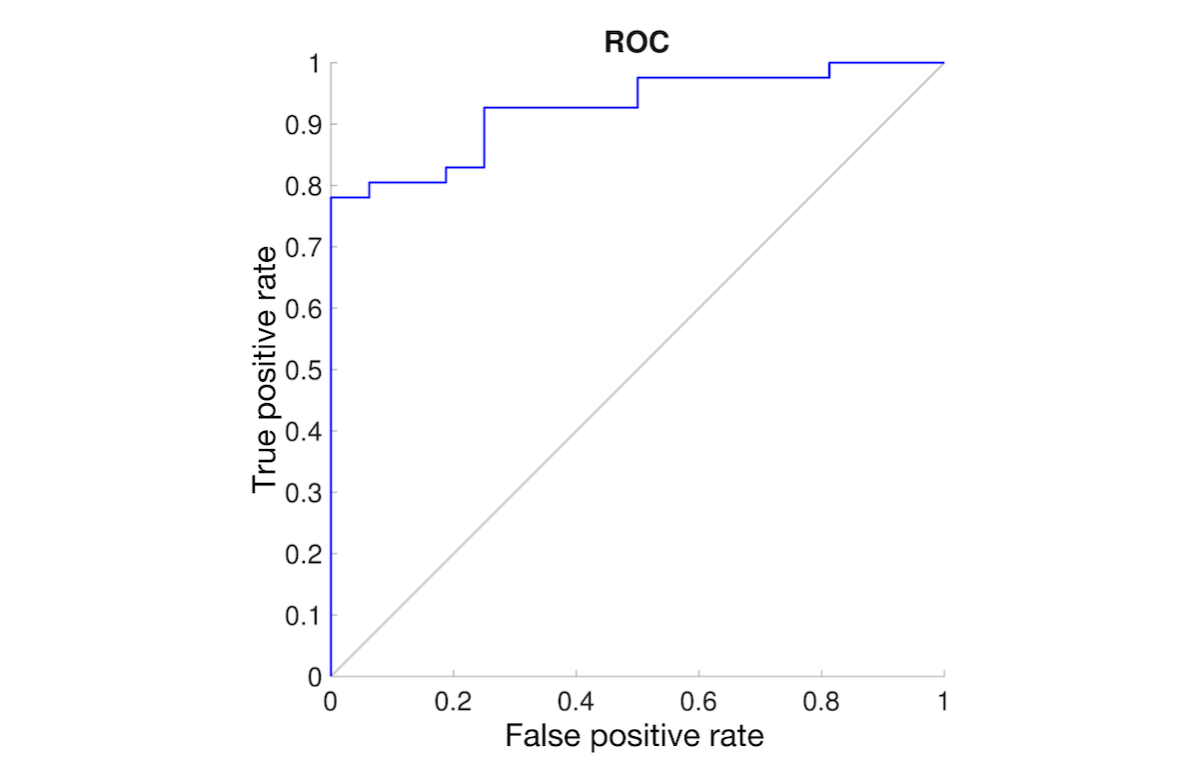
Receiver operating characteristic (ROC) curve for comparison between the Saint Louis University Mental Status (SLUMS) test (cutoff 21) and the BrainCheck composite score.

Finally, we examined the sensitivity and specificity of the BrainCheck tests. Using the physician diagnosis, we found a sensitivity of 89% and a specificity of 78% (see Figure 9). Using a cutoff of 21 on the SLUMS test as the diagnostic criteria, we found a sensitivity of 81% and a specificity of 94% (see Figure 10) [8]. Taken together, these results show that the BrainCheck battery can function as a sensitive and specific screening tool for cognitive impairment.

## Discussion

### Principal Findings

We found that BrainCheck’s composite score is a valid screening tool for cognitive impairment in older adults, as it significantly correlates with scores on the SLUMS test, the MMSE, the MoCA, and physician diagnosis. Unlike the MoCA, the SLUMS exam, and the MMSE, which assess only a few cognitive domains across a series of 12, 11, and 12 items, respectively, BrainCheck’s six assessments are able to measure multiple domains while remaining time-efficient [15], with completion times averaging approximately 21 minutes.

Although individual assessment correlations were only weak to moderate in strength, BrainCheck’s strong composite score correlation, coupled with sensitivities and specificities comparable to those of the commonly used reference tests, demonstrate the value of utilizing the entire battery as a diagnostic aid. Automated scoring and the ability to take BrainCheck without a test administrator reduces potential interviewer bias and variances in physician provision of paper-based tools, which can be affected by training and time pressures in face-to-face assessment of patients. BrainCheck completion time indicates time spent by the subject, not the physician. While somewhat longer than the 10-15-minute estimate of MMSE administration time noted by the publisher of that screening tool, the BrainCheck protocol automates test administration and scoring, reserving physician time to interpretation of results and medical decision making.

Additionally, BrainCheck’s portability, ease-of-use, cost-efficiency, and its ability to store information and connect to electronic medical records should make it a valuable clinical tool. Use of standardized cognitive tests additionally may provide additional physician reimbursement opportunities. Use of brief cognitive screening tools provided during the patient interview are often considered to be elements of the face-to-face visit and are not separately billed and reimbursed.

### Limitations

Geographic and age-dependent convenience sampling was used to create our study sample. As such, availability of participants was limited, restricting sample size. Moreover, the four-to-one gender distribution of our sample exceeds the female-to-male ratios in the general population [26,27]. Lastly, some participants were unable to complete BrainCheck’s entire battery of assessments. While this was accounted for during analysis, the missing data may have limited statistical power. In addition, other screening methods may be necessary for individuals with visual impairment, illiteracy, or movement disorders that preclude administration via a tablet.

Our exploratory physician diagnosis substudy revealed strong correlations between physician assessment and BrainCheck scores. However, due to our small sample size, more research is needed to compare and validate BrainCheck against physician diagnosis.

### Conclusions

Future research should aim to investigate further the potential of BrainCheck to identify not only demented individuals, but those who might be categorized with MCI. A tool with the ability to detect MCI holds great relevance for the future of aging care, as MCI is a common precursor to further cognitive decline. Therefore, detecting MCI may aid primary prevention efforts [7], as well as aiding in the assessment and intervention of treatable or reversible cognitive impairment, potentially prolonging the quality of life of patients and their caregivers. Focus on screening for MCI may additionally reduce the proportion of test takers unable to use a self-administered tool, which can limit utility for individuals with more advanced dementias. Additional study of practice workflow and electronic health record integration will also evaluate factors that may facilitate or inhibit adoption of technology-based assessment tools such as BrainCheck, as physicians balance the need for comprehensive assessment of at-risk individuals with the time pressures of contemporary practice.

## Abbreviations

ADHD: attention deficit hyperactivity disorder
c_a_: contribution to the composite score for each assessment
MCI: mild cognitive impairment
MMSE: Mini-Mental State Examination
MoCA: Montreal Cognitive Assessment
ROC: receiver operating characteristic
s_a_: scaled score for each assessment
SLUMS: Saint Louis University Mental Status exam
TMT: Trail Making Test
w_a_: weights
